# Effect of the *ACAA1* Gene on Preadipocyte Differentiation in Sheep

**DOI:** 10.3389/fgene.2021.649140

**Published:** 2021-06-21

**Authors:** Yanli Wang, Xin Li, Yang Cao, Cheng Xiao, Yu Liu, Haiguo Jin, Yang Cao

**Affiliations:** ^1^Institute of Animal Biotechnology, Jilin Academy of Agricultural Science, Changchun, China; ^2^Institute of Animal Husbandry and Veterinary, Zhejiang Academy of Agricultural Sciences, Hangzhou, China

**Keywords:** *ACAA1* gene, preadipocyte differentiation, sheep, lipid metabolism, adipogenesis

## Abstract

Acetyl-CoA acyltransferase 1 (*ACAA1*) functions as a key regulator of fatty acid β-oxidation in peroxisomes by catalyzing the cleavage of 3-ketoacyl-CoA to acetyl-CoA and acyl-CoA, which participate in the extension and degradation of fatty acids. Thus, *ACAA1* is an important regulator of lipid metabolism and plays an essential role in fatty acid oxidation and lipid metabolism. Our previous study findings revealed that *ACAA1* is closely associated with the peroxisome proliferator-activated receptor (*PPAR*) signaling and fatty acid metabolism pathways, which are involved in fat deposition in sheep, leading to our hypothesis that *ACAA1* may be involved in fat deposition by regulating lipid metabolism. However, the associated molecular mechanism remains unclear. In the present study, to assess the potential function of *ACAA1* in sheep preadipocyte differentiation, we knocked down and overexpressed *ACAA1* in sheep preadipocytes and evaluated the pattern of *ACAA1* gene expression during preadipocyte differentiation by qRT-PCR. *ACAA1* was significantly expressed in the early stage of adipocyte differentiation, and then its expression decreased. *ACAA1* deficiency increased lipid accumulation and the triglyceride content and promoted sheep preadipocyte differentiation, whereas *ACAA1* overexpression inhibited adipogenesis and decreased lipid accumulation and the triglyceride content. Simultaneously, we demonstrated that *ACAA1* deficiency upregulated the expressions of the adipogenic marker genes *PPAR*γ and *C/EBP*α in sheep preadipocytes, but *ACAA1* overexpression inhibited the expressions of these markers, indicating that *ACAA1* affects lipid metabolism by regulating adipogenic marker genes. Our results may promote a better understanding of the regulation of adipogenesis by *ACAA1*.

## Introduction

Adipose tissue, which comprises brown and white adipocytes, is essential for maintaining energy and metabolic homeostasis in animals and plays an essential role in animal endocrine function (Gesta et al., 2007; Galic et al., 2010). Furthermore, adipose tissue secretes several cytokines and hormones that affect the function of cells and tissues (Galic et al., 2010). In mammals, the normal that cleaves peroxisom development of adipose tissue is crucial to maintaining good health. The intramuscular fat content directly affects the meat quality and flavor in animal production, and the abnormal metabolism of adipose tissue can affect meat quality in livestock (Wood et al., 2008). Therefore, elucidating the molecular mechanisms involved in adipocyte differentiation is vital for disease treatment and improving the economic benefits of animal husbandry production.

Acetyl-coenzyme A acyltransferase (ACAA) is a thiolytic enzyme of the acyl-CoA superfamily of metabolic enzymes (Stim-Herndon et al., 1995; Vishwakarma et al., 2013; Ya, 2013). ACAA family members include *ACAA1* and *ACAA2*. *ACAA1*, also known as peroxisome 3-ketoacyl-CoA thiolase, is a crucial enzyme that regulates the β-oxidation of fatty acids in peroxisomes and plays an essential role in fatty acid metabolism (Wanders et al., 2001). The β-oxidation of fatty acids alters the fatty acid content of animals, affecting the meat quality of livestock and poultry.

Because of the changing dietary habits of individuals and the gradual strengthening of healthcare awareness, mutton has become favored by the general public as a green food because of its high protein content, low cholesterol levels, and excellent nutritional qualities (Cao, 2017). The meat quality of animals is closely associated with their fat content because an appropriate intramuscular fat content can increase marbling, reduce shear force, and increase meat flavor and palatability. Additionally, the intramuscular fat content has special significance for production efficiency and meat quality. Intramuscular fat is regulated by lipid metabolism and is synthesized from glycerol and fatty acids. The whole metabolic process is a complex and highly coordinated process (Fang, 2017). Genetic factors affect the accumulation of intramuscular fat by regulating *de novo* lipogenesis and adipocyte differentiation. In a previous screen for functional genes associated with lipid metabolism that affect meat quality, *ACAA2*, a member of the acetyl-CoA acyltransferase family, was identified as a key functional gene affecting lipid metabolism (Li, 2008). *ACAA1* is an important regulator of fatty acid metabolism (Wanders et al., 2001); presently, studies on *ACAA1* have primarily focused on human metabolic diseases, tumors, and cancer. As a regulator of lipid metabolism, few reports have investigated the involvement of *ACAA1* in animal meat quality breeding. In the present study, the pattern of *ACAA1* gene expression during the adipogenic differentiation of sheep preadipocytes was analyzed to assess its role in the adipogenic differentiation process. Investigating the effect of *ACAA1* gene expression on adipogenic differentiation at the cellular and molecular levels is crucial to further improve mutton quality.

## Materials and Methods

### Experimental Animals

Three-month-old lambs were provided by the Institute of Animal Biotechnology (Jilin Academy of Agricultural Science, Changchun, China). All the experimental procedures were examined and approved by the Animal Welfare and Ethics Committee of Jilin Academy of Agricultural Sciences (AWEC 2019A05, May 16, 2019) and complied with the rules and regulations established by the Chinese Experimental Animal Ethics Committee.

### Cell Isolation, Cultivation, and Adipocyte Differentiation

The precursor adipocytes were separated by the collagenase digestion method (Church et al., 2014). Sheep preadipocytes were isolated from the inguinal subcutaneous adipose tissue of 3-month-old male lambs. Next, the animals were anesthetized by air embolization and slaughtered in a specific slaughtering chamber; immediately after slaughter, their adipose tissue was collected under sterile conditions. Freshly isolated subcutaneous adipose tissues were cut into small pieces and weighed. Next, two volumes of 0.2% collagenase II (Sangon Biotech, Shanghai, China) were added per gram of tissue, and the sample was thoroughly mixed. The sample was digested in a water bath at 37°C for 40–90 min. After the digestion was terminated, the cells were obtained by filtration using 200- and 400-mesh filters, and the filtrate was centrifuged three times for 5 min at 1,500 rpm. Subsequently, the cells were cultured in Dulbecco’s modified Eagle’s medium (DMEM/F12; 1:1; Gibco, Shanghai, China) containing 10% fetal bovine serum (Gemini, Woodland, CL, United States) and 1% penicillin/streptomycin (Sangon Biotech) for 24 h at 37°C under an atmosphere with 5% CO_2_. The non-adherent cells were washed away with phosphate-buffered saline (PBS; Gibco, Shanghai, China), and the buffered medium was changed every 48 h. Once the cell confluence reached 100% (0 days), the sheep preadipocytes were induced to differentiate by adding 10 μg/ml of insulin, 0.5 mM 3-isobutyl-1-methylxanthine (IBMX; Sigma, St. Louis, MO, United States) and 1.0 μM dexamethasone (DEX; Sigma) to the culture medium. The cells were then incubated for 2 days, after which 10 μg/ml of insulin (Sigma) was added and the cells were incubated for an additional 2 days (total of 4 days of incubation). Subsequently, the medium was changed every 48 h until the differentiation process was complete (after 8 days).

### Oil Red O Staining and Detection of the Triglyceride Content

Differentiated adipocytes were washed three times with PBS and then fixed with 4% paraformaldehyde (Sangon Biotech, Shanghai, China) for 30 min at 37°C. Next, the cells were washed three times with PBS, stained with 2% oil red O (Sangon Biotech, Shanghai, China) solution at 37°C for 30 min, and washed three times with PBS before being visualized with a microscope for analysis. Subsequently, the oil red O solution was extracted with 100% isopropanol for 10 min, after which the absorbance of lipid droplets at 490 nm was measured on a microboard reader (Ramírezzacarías et al., 1992) (Thermo Fisher Scientific, Carlsbad, CA, United States). The triglyceride content in adipocytes was determined at room temperature using a triglyceride enzyme-linked assay kit E1030 (Applygen, Beijing, China).

### Transfection of Sheep Preadipocytes

Cell transfection was performed as previously reported by Zhang et al. (2018) and Yun et al. ‘(2018). Both the recombinant overexpression plasmid ACAA1-PEX-4 (50 μg) and the empty vector PEX-4 (50 μg) used in the present study were obtained from GenePharma Company (Suzhou, China). ACAA1–PEX-4 was synthesized from PEX-4 and complementary DNA (cDNA) harboring the *ACAA1* gene, both of which were digested with the *Xho*I and *Eco*RI restriction endonucleases. PEX-4 was used as a negative control (NC), and a vector containing enhanced green fluorescent protein (EGFP) was used to confirm the transfection efficiency. Sheep preadipocytes were transfected with 4 μg of the *ACAA1* overexpression plasmid (or NC plasmid) using 10 μl of FuGene HD Transfection Reagent (Promega, Madison, WI, United States) after reaching approximately 70–80% confluence in six-well plates, according to the manufacturer’s instructions. Next, the cells were incubated for 24 h, after which the liquid was exchanged for fresh medium and the cells were induced for 48 h after transfection.

siRNA-ACAA1 (20 μM) and siRNA-NC (NC) were generated by GenePharma. siRNA-NC, as an NC, showed no homology with the target gene. Preadipocytes were cultured to 80% confluency in six-well plates and transfected with 10 μl of siRNA-ACAA1 (or NC) using 5 μl of Lipofectamine 2000 (Invitrogen, Carlsbad, CA, United States) according to the manufacturer’s instructions. The cells were incubated for 8 h, transferred to fresh medium, and then induced 48 h after transfection. The small interfering RNA (siRNA) sequence information is shown in Table 1.

**TABLE 1:** siRNA sequences.

**Symbols**	**siRNA primer sequences (5′–3′)**
siRNA-ACAA1	F: GCUGAGCGGUUUGGCAUUUTT R: AAAUGCCAAACCGCUCAGCTT
siRNA-NC	F: UUCUCCGAACGUGUCACGUTT R: ACGUGACACGUUCGGAGAATT

### Quantitative Real-Time PCR Analysis

Fifteen samples from the normal differentiation period (0, 2, 4, 6, and 8 days, three repeats in each group), 18 samples from the overexpression group and the control group (0, 4, and 8 days, three repeats in the over-ACAA1 and over-NC groups), and 18 samples from the knockdown group and the control group (0, 4, and 8 days, three repeats in the siRNA-ACAA1 and siRNA-NC groups) were collected. Total RNA was isolated using TRIzol reagent (Invitrogen) and reverse transcribed to cDNA using a reverse transcription kit (TaKaRa, Shiga, Japan). Using the synthesized cDNA as a template, quantitative real-time polymerase chain reaction (qRT-PCR) was performed with a LightCycler^®^ 480 II system (Roche) using SYBR Green I Master (Roche), with β*-actin* used as the internal control gene. The relative expression levels of the assayed genes were detected using the 2^–ΔΔCt^ method (Yang et al., 2018; Yun et al., 2018). The primer information is provided in Table 2.

**TABLE 2:** Primers used for quantitative real-time polymerase chain reaction.

**Genes**	**Sequence (5′–3′)**	**Product size (bp)**	**GenBank accession number**
*ACAA1*	F: TCAGGCTGTGTACTGTGTGGA R: AGCGTGATGACCTGTCGAG	120	XM_004018227.3
*PPAR*γ	F: CCGTGGACCTTTCTATGATGG R: TACAGGCTCCACTTTGATTGC	194	NM_001100921.1
*C/EBP*α	F: AAGCCAAGAAGTCCGTGGAC R: AGCACCTTCTGTTGCGTCTCC	127	NM_001308574.1
β*-actin*	F: CCCTGGAGAAGAGCTACGAG R: GGTAGTTTCGTGAATGCCGC	131	U39357.2

### Western Blot Analysis

Fifteen samples were collected from the normal differentiation period (0, 2, 4, 6, and 8 days, three repeats in each group), 12 samples were collected from the overexpression and control groups (4 and 8 days, three repeats in the over-ACAA1 and over-NC groups), and 12 samples were collected from the knockdown and control groups (4 and 8 days, three repeats in the siRNA-ACAA1 and siRNA-NC groups). The cells were washed three times with cold PBS, lysed with a protein lysis buffer (RIPA; Thermo Fisher Scientific) containing protease inhibitors (protease and phosphatase inhibitor cocktail), and then placed on ice for 2 min. Subsequently, the lysate was collected in a 1.5-ml centrifuge tube and placed on ice. The tube was lightly mixed every 5 min; after 30 min, the soluble proteins were obtained by centrifugation at 4°C at 12,000 rpm for 15 min. The protein concentration was determined using an enhanced bicinchoninic acid (BCA) protein detection kit (Beyotime, Shanghai, China). The protein samples were denatured at 95°C for 10 min, and then 20 μg protein sample was separated by sodium dodecyl sulfate–polyacrylamide gel electrophoresis (SDS-PAGE) on a 10% gel before being transferred to polyvinylidene difluoride (PVDF) membranes (Millipore, Burlington, MA, United States). The membranes were washed for 5 min with Tris-buffered saline containing Tween (TBST) and then blocked using 5% skimmed milk at room temperature for 2 h. Subsequently, the membranes were washed three times (5 min each) with TBST and then incubated with a primary antibody overnight at 4°C on a shaker. Next, the membranes were incubated with a corresponding secondary antibody [anti-rabbit or anti-mouse IgG horseradish peroxidase (HRP)-linked, 1:2,000 dilution; Cell Signaling Technology, Danvers, MA, United States] for 90 min at room temperature. The membranes were then washed three times with TBST, after which the immunoreactive bands were visualized using a ChemiScope 6000 Touch (Clinx, Shanghai, China) with an ECL-Plus kit (Applygen) (Yun et al., 2018). The band intensities were estimated by density measurements and normalized to β-actin using Clinx Chemi analysis software. Details regarding the primary antibodies used in the present study are provided in Table 3.

**TABLE 3:** Antibodies used for Western blot analysis.

**Antibodies**	**Source, cat. no.**	**Species raised in mono-/polyclonal**
*ACAA1*	Bioss, cat. no. bs-12566R	Rabbit polyclonal antibody
*PPAR*γ	Bioss, cat. no. bs-4590R	Rabbit polyclonal antibody
*C/EBP*α	Bioss, cat. no. bs-1630R	Rabbit polyclonal antibody
β*-actin*	Abcam, cat. no. ab8224	Mouse monoclonal antibody

### Statistical Analysis

The qRT-PCR results were statistically analyzed using the 2^–ΔΔCt^ method, and the gray values of the protein were analyzed using Clinx image analysis software. All the data are presented as the mean ± standard error of three independent experiments performed in triplicate. Calculations and figures were produced by one-way ANOVA using GraphPad Prism version 6.00 for Windows. The statistical significance levels were set at *p* < 0.05 (Yang et al., 2018).

## Results

### *ACAA1* Expression Pattern During Sheep Preadipocyte Differentiation

**FIGURE 1:**
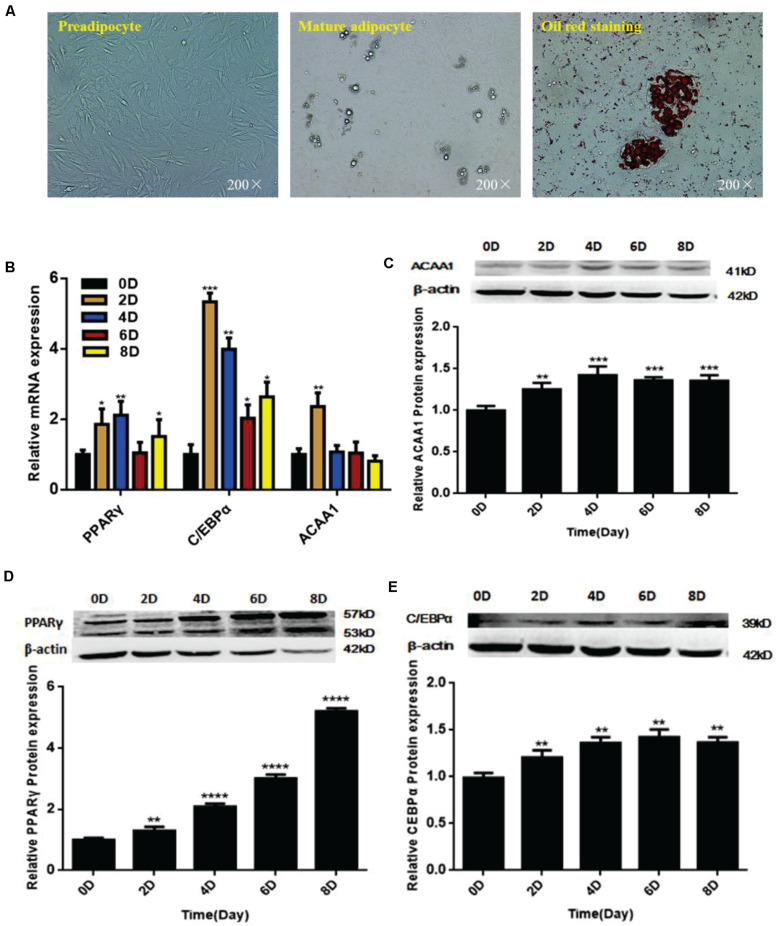
*ACAA1* and adipogenic marker gene expression during sheep preadipocyte differentiation. **(A)** Sheep preadipocytes, mature adipocytes, and oil red O staining (200×). **(B)** Expression patterns of adipogenic marker genes (*PPAR*γ and *C/EBP*α) in differentiating sheep adipocytes. **(C)** *ACAA1* protein expression in differentiating sheep adipocytes was determined by Western blot analysis. **(D,E)** The protein expression levels of *PPAR*γ and *C/EBP*α during adipocyte differentiation were evaluated on days 0, 2, 4, 6, and 8. Densitometric analyses of the Western blots. With the differentiation of cells, the expression levels of adipogenic genes gradually increased (*p < 0.05, **p < 0.01, ***p < 0.001, and ****p < 0.0001). *ACAA1*, acetyl-CoA acyltransferase 1; *PPAR*γ, peroxisome proliferator-activated receptor γ; *C/EBP*α, CCAAT/enhancer binding protein α.

We isolated and used sheep preadipocytes as a model of adipogenesis to assess the associated changes in *ACAA1* expression. Sheep preadipocytes were stimulated to differentiate for 8 days using the “cocktail” method. The sheep preadipocytes exhibited obvious morphological changes after 8 days of differentiation and contained many cytoplasmic lipid droplets showing positive staining for oil red O, where typical round lipid droplets and smaller lipid droplets gathered into large lipid droplets were observed, all of which are typical characteristics of mature adipocytes (Figure 1A). The messenger RNA (mRNA) and protein levels of adipogenic markers (*PPAR*γ and *C/EBP*α) were analyzed during sheep preadipocyte differentiation by qRT-PCR and Western blotting, respectively. During adipogenesis, the expression of adipogenic markers increased with the lipid accumulation in sheep adipocytes (Figures 1B,D,E), indicating that the isolated sheep preadipocytes could be used in subsequent experiments. Interestingly, the *ACAA1* mRNA expression levels were significantly increased in the first 2 days of sheep adipocyte differentiation and then gradually decreased (Figure 1B), while the *ACAA1* protein levels increased in the early stage of differentiation (0–4 days) before decreasing (Figure 1C). The *ACAA1* mRNA and protein expression trends were similar to those observed for the adipogenic marker *C/EBP*α. Adipogenesis involves the dynamic regulation of gene expression, and these results suggest that *ACAA1* may be involved in regulating sheep preadipocyte differentiation.

### *ACAA1* Knockdown Promotes Preadipocyte Differentiation

#### *ACAA1* Knockdown Increases Lipid Accumulation and the Triglyceride Content

**FIGURE 2:**
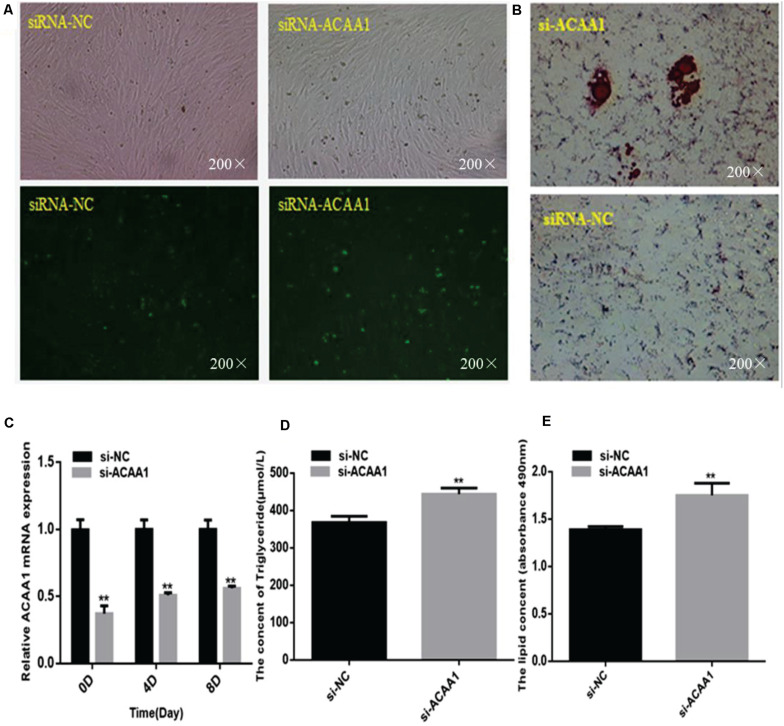
*ACAA1* knockdown increases lipid accumulation and the triglyceride content during sheep preadipocyte differentiation. **(A)** Determination of the transfection efficiency of siRNA-ACAA1 (200×). **(B)** After 8 days of differentiation, the cellular lipid droplets were stained with oil red O (200×). **(C)** *ACAA1* mRNA expression was detected after siRNA-ACAA1 or NC transfection on days 0, 4, and 8 of adipogenic differentiation. The siRNA-ACAA1 groups all showed significantly lower expressions than the control group (**p < 0.01). **(D)** The triglyceride content after 8 days of induction in the siRNA-ACAA1 group was significantly higher than that in the control group (**p < 0.01). **(E)** The lipid droplet content measured at 490 nm after 8 days of induction was significantly higher in the siRNA-ACAA1 group than that in the control group (**p < 0.01). *siRNA*, small interfering RNA; *NC*, negative control.

**FIGURE 3:**
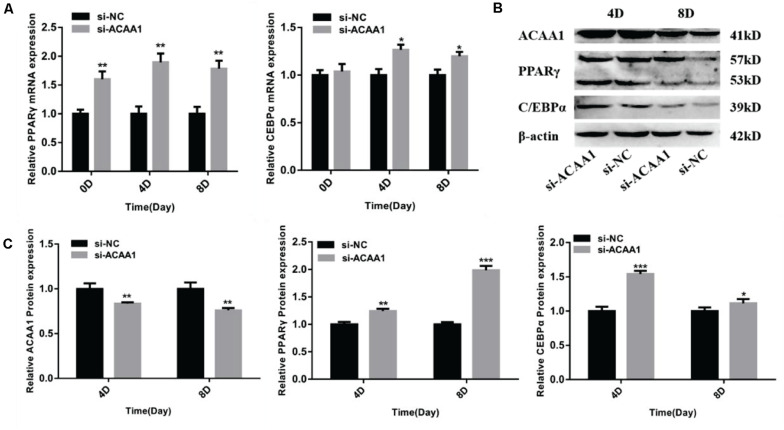
*ACAA1* knockdown regulates adipogenic marker gene expression. **(A)** Sheep preadipocytes were transfected with siRNA-ACAA1 or siRNA-NC and differentiated into mature adipocytes. The mRNA expression levels of adipogenic marker genes (*PPAR*γ and *C/EBP*α) were evaluated on days 0 (after cell amalgamation), 4, and 8 of adipogenic differentiation. On day 0 of adipogenic differentiation, the mRNA expression levels of the marker genes were higher than those of the control group, but only *PPAR*γ reached a significant level (**p < 0.01). On days 4 and 8 of adipogenic differentiation, the mRNA expression levels of *PPAR*γ and *C/EBP*α were significantly higher than those of the control group (*p < 0.05 and **p < 0.01). **(B)** Protein expression levels of *ACAA1* and adipogenic marker genes on days 4 and 8 of adipogenic differentiation. *Columns 1* and *2* are the siRNA-ACAA1 processing group and the siRNA-NC processing group, respectively (day 4). *Columns 3* and *4* are the siRNA-ACAA1 processing group and the siRNA-NC processing group, respectively (day 8). β*-actin* is the internal reference gene. **(C)** The protein expression levels of *ACAA1* and adipogenic marker genes were assessed on days 4 and 8 of adipogenic differentiation. The protein expression levels of *ACAA1* were significantly lower than those of the control group (**p < 0.01), and the protein expression levels of *PPAR*γ and *C/EBP*α were significantly higher than those of the control group (*p < 0.05, **p < 0.01, and ***p < 0.001). *NC*, negative control.

To assess whether *ACAA1* affects sheep preadipocyte differentiation, we knocked down *ACAA1* expression in sheep preadipocytes by transfecting siRNA-ACAA1 or siRNA-NC into sheep preadipocytes cultured to 80% confluency. The *ACAA1* mRNA and protein expression levels in the knockdown group were markedly downregulated compared with those in the NC group (*p* < 0.01; Figures 2A,C, 3B). Oil red O staining and lipid droplet extraction analyses demonstrated that more lipids accumulated in adipocytes from the *ACAA1* knockdown group than from the NC group (*p* < 0.01; Figures 2B,E), and the triglyceride content of the knockdown group was also significantly greater (*p* < 0.01; Figure 2D).

#### *ACAA1* Knockdown Enhances Adipogenic Marker Gene Expression

The mRNA expression levels of adipogenic marker genes (including *PPAR*γ and *C/EBP*α) involved in differentiation were measured on days 0, 4, and 8 after induction (Figure 3A), while the protein expression levels were measured 4 and 8 days later (Figure 3B). The mRNA expressions of the adipogenic marker genes were upregulated following *ACAA1* knockdown (*p* < 0.05 and *p* < 0.01; Figure 3A), while the PPARγ and C/EBPα protein expressions were significantly increased by *ACAA1* deficiency (*p* < 0.05, *p* < 0.01, and *p* < 0.001; Figure 3C). Thus, the *ACAA1* gene may be a negative regulator of adipogenic differentiation.

### *ACAA1* Overexpression Inhibits Preadipocyte Differentiation

#### *ACAA1* Overexpression Decreases Preadipocyte Lipid Accumulation and the Triglyceride Content

**FIGURE 4:**
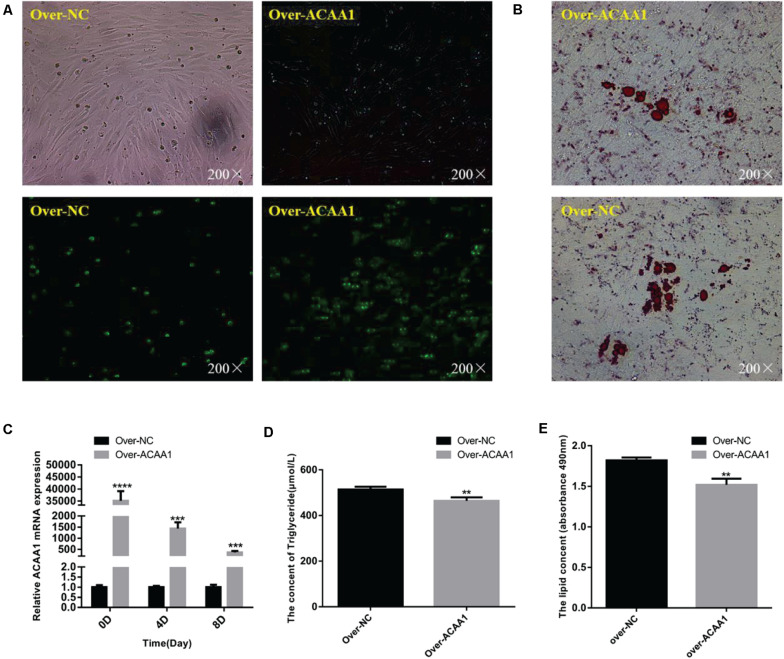
*ACAA1* overexpression decreases lipid accumulation and the triglyceride content in preadipocytes. **(A)** Determination of the transfection efficiency of the *ACAA1* overexpression plasmid (200×). **(B)** After 8 days of differentiation, the cellular lipid droplets were stained with oil red O (200×). **(C)** *ACAA1* mRNA expression was assessed after cells were transfected with the *ACAA1* overexpression or the NC plasmids on days 0, 4, and 8 of adipogenic differentiation. The expression in the over-ACAA1 group was significantly higher than that in the control group (****p < 0.01 and ***p < 0.001). The difference was largest in the early stage of cell differentiation. **(D)** After 8 days of induction, the triglyceride content in the over-ACAA1 group was significantly lower than that in the control group (**p < 0.01). **(E)** The lipid droplet content measured at 490 nm after 8 days of induction in the over-ACAA1 group was significantly lower than that in the control group (**p < 0.01).

**FIGURE 5:**
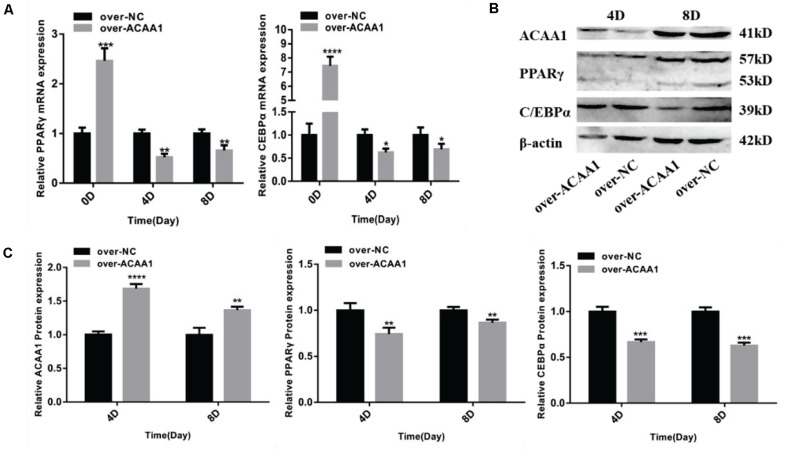
*ACAA1* overexpression regulates adipogenic marker gene expression. **(A)** Sheep preadipocytes were transfected with *ACAA1* overexpression or negative control (NC) plasmids and differentiated into mature adipocytes. The mRNA expression levels of adipogenic marker genes (*PPAR*γ and *C/EBP*α) were measured on days 0, 4, and 8 of adipogenic differentiation. Except day 0, the mRNA expression levels of *PPAR*γ and *C/EBP*α were significantly lower than those of the control group (*p < 0.05, **p < 0.01, ***p < 0.001, and ****p < 0.0001). **(B)** Protein expression levels of *ACAA1* and adipogenic marker genes on days 4 and 8 of adipogenic differentiation. *Columns 1* and *2* are the over-ACAA1 processing and over-NC processing groups, respectively (day 4). *Columns 3* and *4* are the over-ACAA1 processing and over-NC processing groups, respectively (day 8). β*-actin* is the internal reference gene. **(C)** The protein expression levels of *ACAA1* and adipogenic marker genes were assessed on days 4 and 8 of adipogenic differentiation. The protein expression levels of *ACAA1* were significantly higher than those of the control group (****p < 0.0001 and **p < 0.01), and the protein expression levels of *PPAR*γ and *C/EBP*α were significantly lower than those of the control group (**p < 0.01 and ***p < 0.001).

The effects of *ACAA1* overexpression on sheep preadipocyte differentiation were evaluated. The *ACAA1* mRNA and protein expression levels in the overexpression group were significantly higher than those in the NC group (*p* < 0.01; Figures 4A,C, 5B). Oil red O staining and lipid droplet extraction revealed that *ACAA1* overexpression significantly inhibited preadipocyte differentiation and lipid accumulation in sheep adipocytes (*p* < 0.01; Figures 4B,E). Additionally, the triglyceride content in the overexpression group was significantly lower than that in the NC group (*p* < 0.01; Figure 4D).

#### *ACAA1* Overexpression Decreases Adipogenic Marker Gene Expression

*ACAA1* overexpression remarkably downregulated the mRNA levels of the adipogenic marker genes *PPAR*γ and *C/EBP*α on days 4 and 8 (*p* < 0.05, *p* < 0.01, *p* < 0.001, and *p* < 0.0001; Figure 5A). Consistent with these findings, Western blotting showed that *ACAA1* overexpression significantly decreased the protein levels of these adipogenic marker genes (*p* < 0.01 and *p* < 0.001; Figures 5B,C).

## Discussion

A strong correlation exists between the fat content and meat quality in animals, which can affect the sensory indexes of meat, such as tenderness, color, flavor, and nutritional value (Huan-Hsien, 2011). As one determinant of meat quality, fat content has become a research hotspot for livestock and poultry meat quality in recent years. However, presently, studies on the regulation of fat development and deposition have primarily focused on pigs (Zou et al., 2018), cattle (Park et al., 2018), and chickens (Fu et al., 2018), whereas such studies in sheep have been lacking.

Mammalian adipogenesis is a complicated physiological process involving the expressions of multiple genes, signal transduction pathways, and network regulation to balance the proliferation and differentiation of preadipocytes (Gesta et al., 2007). The *ACAA1* gene encodes a thiolytic enzyme that cleaves peroxisomal 3-ketoacyl-CoA into acetyl-CoA and acyl-CoA, which participate in the extension and degradation of fatty acids. *ACAA1* is an important regulatory gene involved in cell lipid metabolism (Wanders et al., 2001) and is a target of biotherapy for human metabolic diseases (Colas et al., 2011; Park et al., 2012; Klimosch et al., 2013; Liu et al., 2015; Nwosu et al., 2018; Zhang et al., 2019). In a previous study, we investigated the transcriptome characteristics associated with the intramuscular fat content of the sheep longissimus dorsi muscle and observed significant differences in the transcriptional levels of *ACAA1*, which is associated with members of the PPAR signaling and fatty acid metabolism signaling pathways involved in fat deposition in sheep. Therefore, we speculated that *ACAA1* may be involved in fat deposition by regulating lipid metabolism.

In the present study, we analyzed the pattern of *ACAA1* gene expression during the differentiation of cultured sheep preadipocytes, and the results suggest that *ACAA1* may be a functional regulator of sheep fat deposition. Several studies have demonstrated that *ACAA1* plays an important role in animal lipid metabolism (Lisowski et al., 2014; Zhang and Wang, 2015; Zhu et al., 2016). For example, *ACAA1* gene upregulation is involved in fatty acid biosynthesis and lipid metabolism in beef cattle (Lisowski et al., 2014), as well as increased liver triglyceride metabolism, lipid decomposition, and fatty acid oxidation in mice fed a high-fat diet, which can reduce intestinal lipid absorption (Zhu et al., 2016). In the present study, to investigate the potential function of *ACAA1* in sheep preadipocyte differentiation, we assessed the effects of *ACAA1* knockdown and overexpression in sheep preadipocytes. After induction, we observed that *ACAA1* deficiency increased lipid accumulation and the triglyceride content and promoted adipogenesis. These results are consistent with those reported for chicken intramuscular preadipocytes, in which the inhibition of *ACAA1* gene expression in fatty acid peroxisomes was shown to promote the deposition of intramuscular fat in chickens (Li et al., 2019). However, *ACAA1* overexpression inhibits adipogenesis and decreases lipid accumulation and the triglyceride content. The upregulation of *ACAA1* expression has been shown to reduce the intramuscular fat content in the longissimus dorsi of large white pigs (Yao, 2020). These results were consistent with previous findings (Xie et al., 2014; Zhang and Wang, 2015; Zhu et al., 2016). *ACAA1* gene upregulation enhanced fatty acid β-oxidation and triglyceride metabolism in the liver and decreased lipid accumulation in animals (Zhang and Wang, 2015). As a key enzyme of lipid metabolism, *ACAA1* may promote fatty acid metabolism and fatty acid degradation by participating in the β-oxidation of very long-chain fatty acids, affecting the function of fat metabolism in obese rats to achieve fat reduction and weight reduction (Meng, 2020). Fenofibrate (FB) significantly reduced abdominal fat and liver lipid accumulation in mice fed a high-fat diet by increasing *ACAA1* mRNA expression (Xie et al., 2014). Taken together, these results suggest that *ACAA1* may be a negative regulator of sheep preadipocyte differentiation and plays a crucial role in sheep fat deposition.

We found that *ACAA1* deficiency upregulates the expression of the adipogenic marker genes *PPAR*γ and *C/EBP*α in sheep preadipocytes, but *ACAA1* overexpression inhibits *PPAR*γ and *C/EBP*α expressions. Interestingly, the mRNA levels of these adipogenic marker genes on day 0 were significantly higher in the *ACAA1* overexpression group than those in the NC group, possibly because of a transcriptional delay effect for the different genes. PPARG is a key transcription factor in adipogenesis involved in maintaining the function of mature adipocytes (Imai et al., 2004; Schupp et al., 2009). The *ACAA1* gene is a downstream target gene of PPAR and is controlled by this regulator (Lake, 1995). In the PPAR signaling pathway, fatty acids serve as ligands that promote PPAR expression. PPAR activation can upregulate the expressions of downstream genes such as *ACAA1*, *ACOX1*, and *CPT-1* and stimulate fatty acid oxidation. Additionally, adipogenic gene upregulation resulting from PPAR activation can promote adipocyte differentiation (Li et al., 2019). *ACAA1* is closely related to the intramuscular fat content in pigs (Wu et al., 2013). The expression of *ACAA1* gene is regulated by the activation of the PPAR signaling pathway, which affects fat deposition and the intramuscular fat content in pigs (Yao, 2020). Based on previous study findings and those described herein, we hypothesize that the possible molecular mechanisms involved in preadipocyte differentiation are as follows. *ACAA1* knockdown reduces the oxidation of fatty acids in the PPAR signaling pathway, increasing the contents of saturated and unsaturated fatty acids in sheep adipocytes. The accumulated fatty acids further stimulate the transcriptional activity of PPAR, after which the expressions of downstream adipogenic genes become upregulated, promoting the differentiation of sheep preadipocytes.

In summary, our results suggest that *ACAA1* may be a transcriptional regulator of *PPAR*γ and *C/EBP*α because of its ability to affect sheep preadipocyte differentiation. *ACAA1* deficiency promoted adipogenesis and lipid accumulation, while *ACAA1* overexpression inhibited sheep preadipocyte differentiation. These findings provide new molecular insights for further studies on the role of *ACAA1* in regulating fat metabolism and improving meat quality in sheep. However, further research is needed to elucidate the specific mechanism of *ACAA1* in regulating adipogenesis. There is still a long way to go for molecular breeding as a functional gene.

## Data Availability Statement

The raw data supporting the conclusions of this article will be made available by the authors, without undue reservation.

## Ethics Statement

The animal study was reviewed and approved by the Animal Welfare and Ethics Committee of Jilin Academy of Agricultural Sciences.

## Author Contributions

YC (seventh author) designed the study. YW, XL, and YC (third author) performed the experiments. CX, YL, and YW analyzed the data. YW wrote the manuscript. HJ and YC (seventh author) corrected and approved the final version of the manuscript. All authors participated in the revision of the manuscript.

## Conflict of Interest

The authors declare that the research was conducted in the absence of any commercial or financial relationships that could be construed as a potential conflict of interest.

## References

[B1] Cao Y. (2017). *Comparative Analysis of Whole Genome Methylation and Transcriptome of the Longissimus Dorsi Muscle of Du Han and Small–Tail Han Sheep.* Changchun: Jilin University.

[B2] Church C. D. Berry R. Rodeheffer M. S. (2014). Isolation and study of adipocyte precursors. *Methods Enzymol.* 537 31–46. 24480340 10.1016/B978-0-12-411619-1.00003-3 PMC4276307

[B3] Colas E. Perez C. Cabrera S. (2011). Molecular markers of endometrial carcinoma detected in uterine aspirates. *Int. J. Cancer*. 129 2435–2444. 10.1002/ijc.25901 21207424

[B4] Fang X. B. (2017). *Identification and Functional Verification of Candidate Genes for Beef Quality Traits Based on Transcriptome and Genome-Wide Methylation Analysis.* Changchun: Jilin University.

[B5] Fu S. Zhao Y. Li Y. Li G. Chen Y. Li Z. (2018). Characterization of miRNA transcriptome profiles related to breast muscle development and intramuscular fat deposition in chickens. *J. Cell. Biochem.* 119 7063–7079. 29737555 10.1002/jcb.27024

[B6] Galic S. Oakhill J. S. Steinberg G. R. (2010). Adipose tissue as an endocrine organ. *Mol. Cell. Endocrinol*. 316 129–139. 19723556 10.1016/j.mce.2009.08.018

[B7] Gesta S. Tseng Y. H. Kahn C. R. (2007). Developmental origin of fat: tracking obesity to its source. *Cell* 131 242–256. 10.1016/j.cell.2007.10.004 17956727

[B8] Huan-Hsien C. (2011). *Study on Molecular Regulatory Network and Related Genes of Intramuscular Fat Formation in Broilers.* Beijing: Chinese Academy of Agricultural Sciences.

[B9] Imai T. Takakuwa R. Marchand S. Dentz E. Bornert J. M. Messaddeq N. (2004). Peroxisome proliferator-activated receptor gamma is required in mature white and brown adipocytes for their survival in the mouse. *Proc. Natl. Acad. Sci. U.S.A*. 101 4543–4547. 10.1073/pnas.0400356101 15070754 PMC384783

[B10] Klimosch S. N. Forstin A. Eckert J. (2013). Functional TLR5 genetic variants affect human colorectal cancer survival. *Cancer Res*. 73 7232–7242. 10.1158/0008-5472.can-13-1746 24154872

[B11] Lake B. G. (1995). Mechanisms of hepatocarcinogenicity of peroxisome-proliferating drugs and chemicals. *Annu. Rev. Pharmacol. Toxicol*. 35 483–507. 10.1146/annurev.pa.35.040195.002411 7598504

[B12] Li G. Fu S. Chen Y. Jin W. Zhai B. Li Y. (2019). MicroRNA-15a regulates the differentiation of intramuscular preadipocytes by targeting *ACAA1*. *ACOX1* and *SCP2* in Chickens. *Int. J. Mol. Sci*. 20:4063. 10.3390/ijms20164063 31434294 PMC6720712

[B13] Li W. J. (2008). *Screening and High-spirited Regulation of functional genes of Chicken related Fat Metabolism.* Beijing: Chinese Academy of Agricultural Sciences.

[B14] Lisowski P. Kościuczuk E. M. Gościk J. Pierzchała M. Rowińska B. Zwierzchowski L. (2014). Hepatic transcriptome profiling identifies differences in expression of genes associated with changes in metabolism and postnatal growth between Hereford and Holstein-Friesian bulls. *Anim. Genet*. 45 288–292. 10.1111/age.12116 24304134

[B15] Liu F. Li H. Chang H. Wang J. Lu J. (2015). Identification of hepatocellular carcinoma-associated hub genes and pathways by integrated microarray analysis. *Tumori* 101 206–214. 10.5301/tj.5000241 25768320

[B16] Meng Y. (2020). *Effects of Hypoxic Training on Liver Lipid Metabolism Related Genes and Serum Exocrine in Obese Rats.* Shandong: Qufu Normal University.

[B17] Nwosu Z. C. Battello N. Rothley M. (2018). Liver cancer cell lines distinctly mimic the metabolic gene expression pattern of the corresponding human tumours. *J. Exp. Clin. Cancer Res*. 37:211. 10.1186/s13046-018-0872-6 PMC6122702 30176945

[B18] Park S. J. Beak S. H. Jung D. J. S. Kim S. Y. Jeong I. H. Piao M. Y. (2018). Genetic, management, and nutritional factors affecting intramuscular fat deposition in beef cattle - A review. *Asian-Australas J. Anim. Sci*. 31 1043–1061. 10.5713/ajas.18.0310 29879830 PMC6039335

[B19] Park S. K. Yang J. J. Oh S. Cho L. Y. Ma S. H. Shin A. (2012). Innate immunity and non-Hodgkin’s lymphoma (NHL) related genes in a nested case-control study for gastric cancer risk. *PLoS One* 7:e45274. 10.1371/journal.pone.0045274 23028900 PMC3448653

[B20] Ramírezzacarías J. L. Castromuñozledo F. Kuriharcuch W. (1992). Quantitation of adipose conversion and triglycerides by staining intracytoplasmic lipids with oil red O. *Histochemistry* 97 493–497. 10.1007/bf00316069 1385366

[B21] Schupp M. Cristancho A. G. Lefterova M. I. (2009). Re-expression of GATA2 cooperates with peroxisome proliferator-activated receptor-gamma depletion to revert the adipocyte phenotype. *J. Biol. Chem*. 284 9458–9464. 10.1074/jbc.m809498200 19136559 PMC2666598

[B22] Stim-Herndon K. P. Petersen D. J. Bennett G. N. (1995). Characterization of an acetyl-CoA C-acetyltransferase (thiolase) gene from Clostridium acetobutylicum ATCC 824. *Gene* 154 81–85. 10.1016/0378-1119(94)00838-j 7867955

[B23] Vishwakarma R. K. Ruby, Singh S. Sonawane P. D. Srivastava S. Kumari U. (2013). Molecular cloning, biochemical characterization, and differential expression of an Acetyl-CoA C-Acetyltransferase Gene (AACT) of Brahmi (Bacopa monniera). *Plant Mol. Biol. Rep*. 31 547–557. 10.1007/s11105-012-0523-6 PMC3781283 24431524

[B24] Wanders R. J. Vreken P. Ferdinandusse S. Jansen G. A. Waterham H. R. van Roermund C. W. (2001). Peroxisomal fatty acid alpha- and beta-oxidation in humans: enzymology, peroxisomal metabolite transporters and peroxisomal diseases. *Biochem. Soc. Trans.* 29(Pt 2) 250–267. 10.1042/bst0290250 11356164

[B25] Wood J. D. Enser M. Fisher A. V. Nute G. R. Sheard P. R. Richardson R. I. (2008). Fat deposition, fatty acid composition and meat quality: a review. *Meat Sci*. 78 343–358. 10.1016/j.meatsci.2007.07.019 22062452

[B26] Wu T. Zhang Z. H. Yuan Z. Q. Lo L. J. Chen J. Wang Y. (2013). Distinctive genes determine different intramuscular fat and muscle fiber ratios of the longissimus dorsi muscles in jinhua and landrace pigs. *PLoS One* 8:e53181. 10.1371/journal.pone.0053181 23301040 PMC3536781

[B27] Xie W. Zhang S. Lei F. Ouyang X. Du L. (2014). Ananas comosus L. Leaf Phenols and p-Coumaric acid regulate liver fat metabolism by upregulating CPT-1 expression. *Evid. Based Complement Alternat Med*. 2014:90 3258. 10.1155/2014/903258 PMC4145745 25197313

[B28] Ya Y. Y. (2013). *Cloning and Quantitative Expression of 3-Ketoacyl-CoA Thiolase Gene in ISOCHRYSIS Globosa and Analysis of Fatty Acid Content Under Environmental Stress.* Shihezi: Shihezi University.

[B29] Yang Y. Fang X. Yang R. Yu H. Jiang P. Sun B. (2018). MiR-152 regulates apoptosis and triglyceride production in MECs via targeting ACAA2 and HSD17B12 genes. *Sci. Rep*. 8:417. 10.1038/s41598-017-18804-x PMC5765104 29323178

[B30] Yao C. G. (2020). *Identification of Gene Expression Patterns Affecting Porcine Fat Depositionusing GEO Public Database.* Changchun: Jilin University.

[B31] Yun J. Jin H. Cao Y. Zhang L. Zhao Y. Jin X. (2018). RNA-Seq analysis reveals a positive role of HTR2A in adipogenesis in yan yellow cattle. *Int. J. Mol. Sci*. 19:1760. 10.3390/ijms19061760 29899319 PMC6032390

[B32] Zhang B. Wu Q. Wang Z. Xu R. Hu X. Sun Y. (2019). The promising novel biomarkers and candidate small molecule drugs in kidney renal clear cell carcinoma: evidence from bioinformatics analysis of high-throughput data. *Mol. Genet. Genomic Med*. 7:e607. 10.1002/mgg3.607 30793530 PMC6503072

[B33] Zhang L. Y. Wang Y. M. (2015). *Persistent Effects of Intake of Sea Cucumber Phospholipids on the Expression of Genes Related to Lipid Metabolism in Mice. Abstract of the Annual Meeting of Chinese Fisheries Society.* Hangzhou: China Fisheries Society.

[B34] Zhang Y. Wang Y. Wang X. Ji Y. Cheng S. Wang M. (2018). Acetyl-coenzyme A acyltransferase 2 promote the differentiation of sheep precursor adipocytes into adipocytes. *J. Cell. Biochem. [Online ahead of print]* 10.1002/jcb.28080 30485515

[B35] Zhu S. Park S. Lim Y. Shin S. Han S. N. (2016). Korean pine nut oil replacement decreases intestinal lipid uptake while improves hepatic lipid metabolism in mice. *Nutr. Res. Pract*. 10 477–486. 10.4162/nrp.2016.10.5.477 27698954 PMC5037064

[B36] Zou C. Li L. Cheng X. Li C. Fu Y. Fang C. (2018). Identification and functional analysis of long intergenic non-coding RNAs underlying intramuscular fat content in pigs. *Front Genet*. 27:102. 10.3389/fgene.2018.00102 29662503 PMC5890112

