# Supplementary figures and images for: Effect of the ACAA1 Gene on Preadipocyte Differentiation in Sheep
Source: Front Genet. 2021 Jun 21;12:649140. doi: 10.3389/fgene.2021.649140 (PMC8255805; doi:10.3389/fgene.2021.649140)

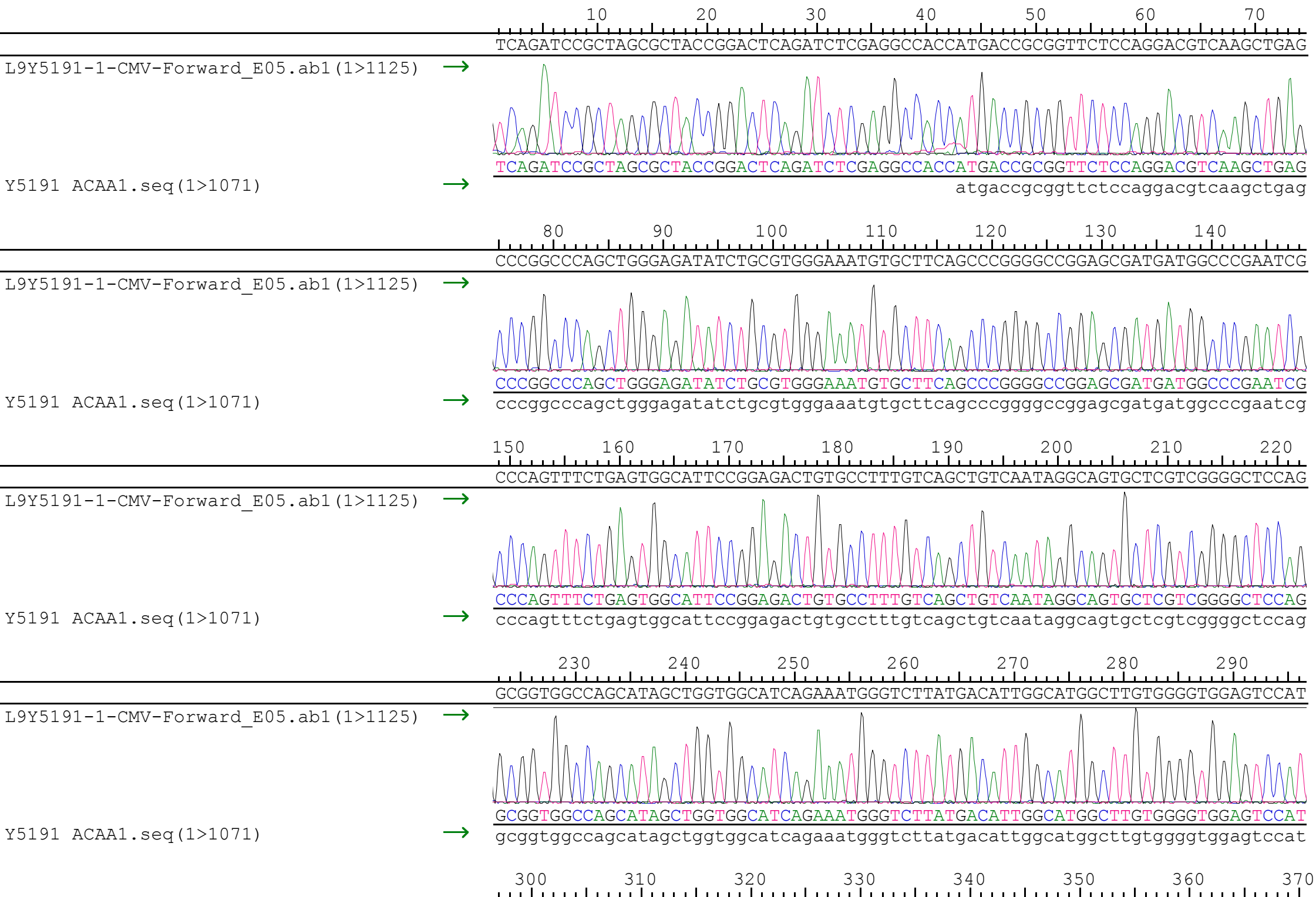

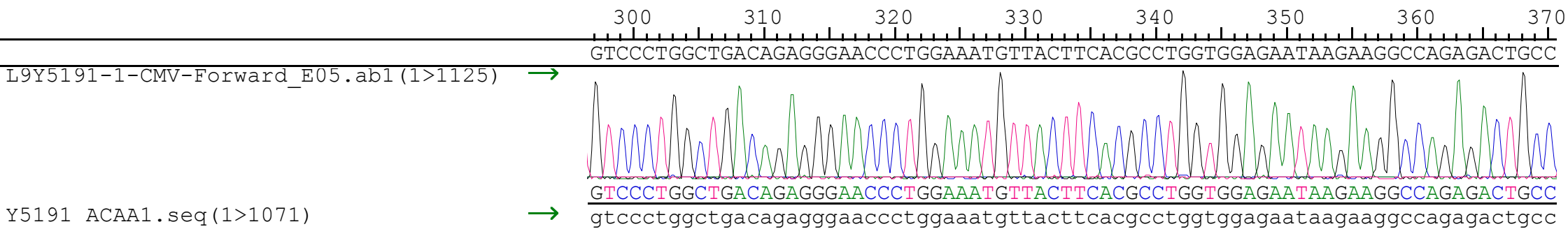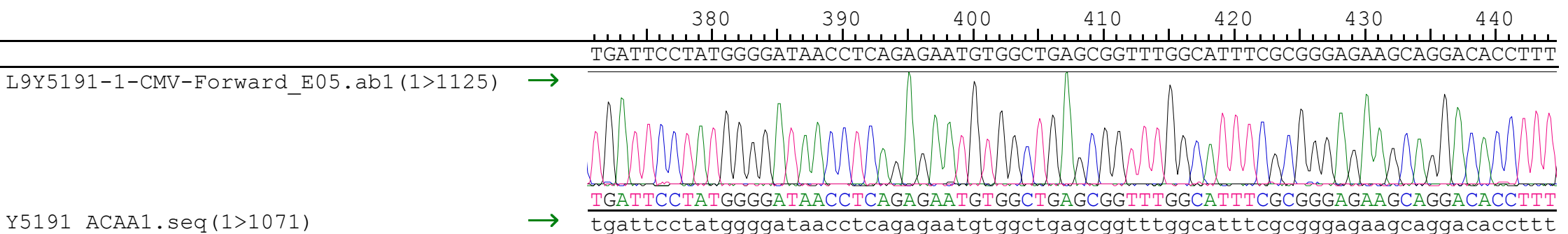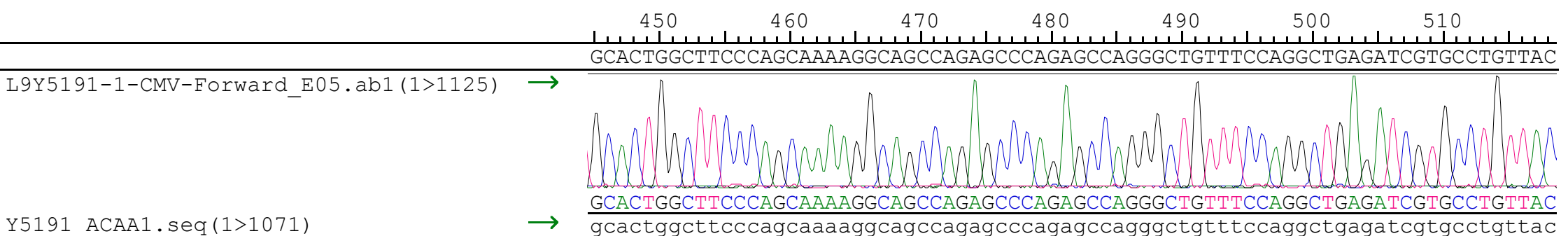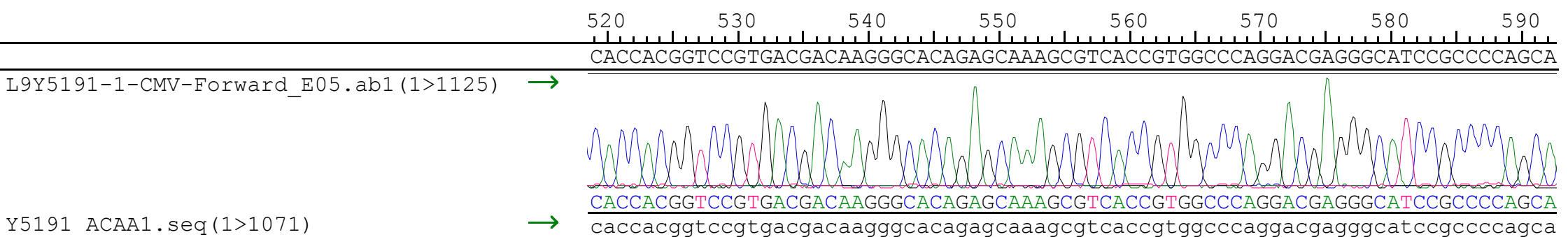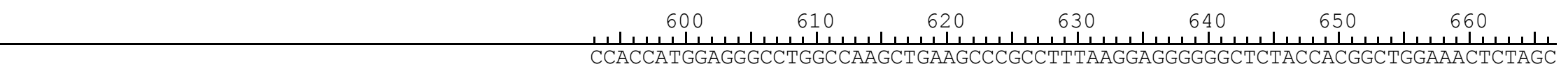

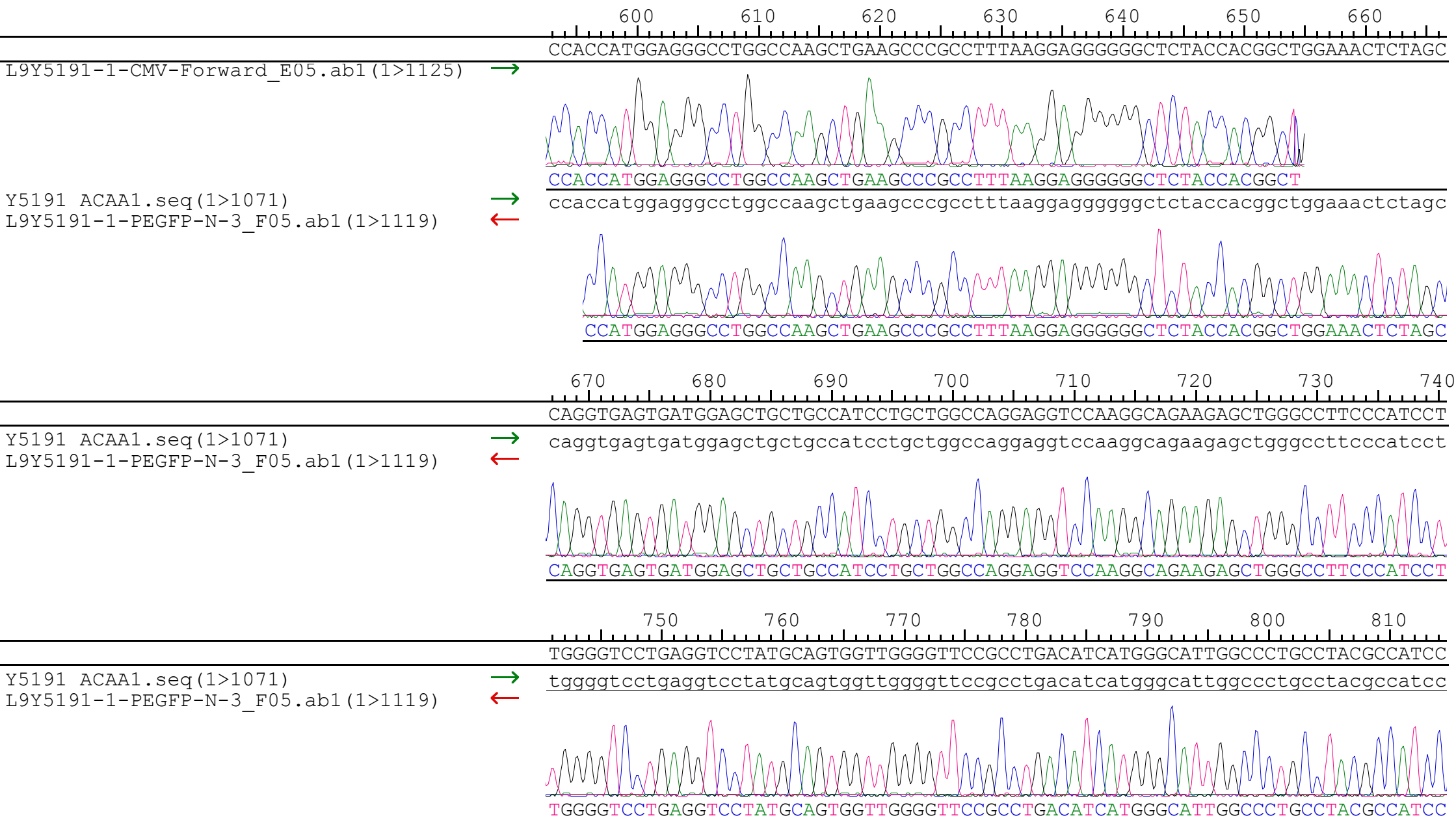

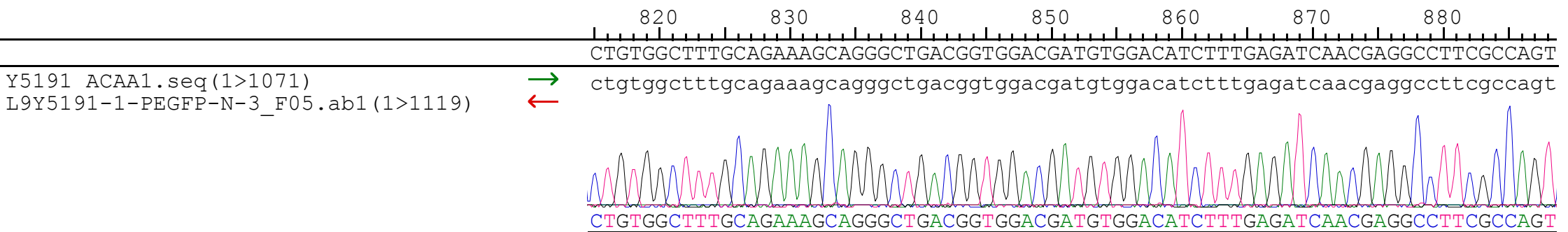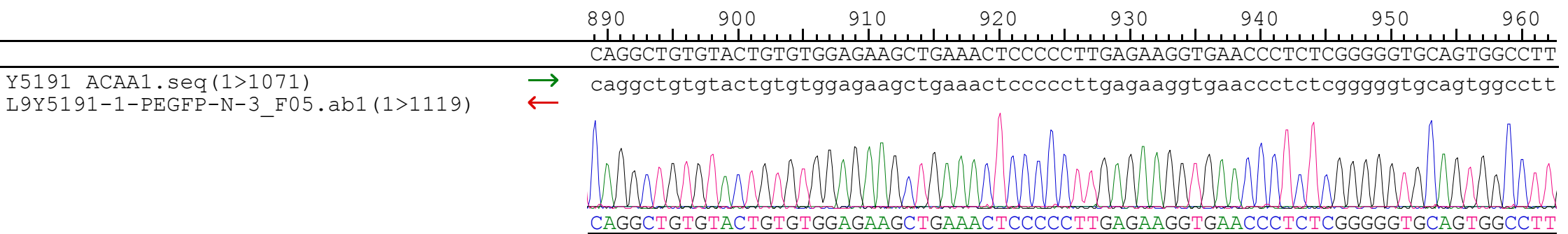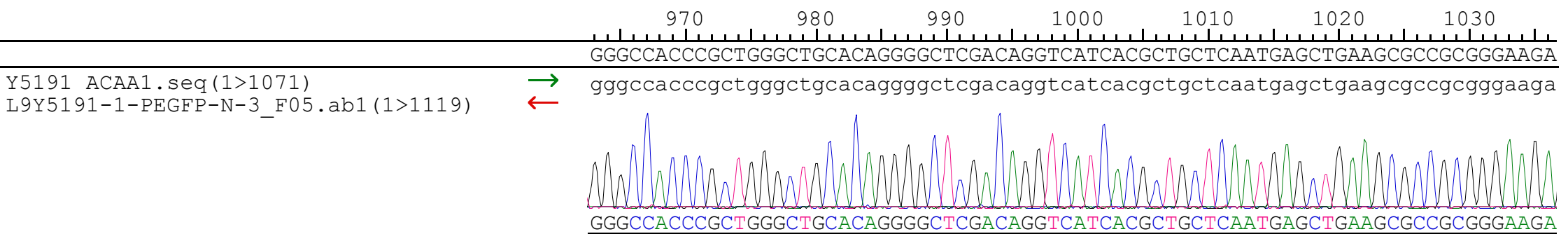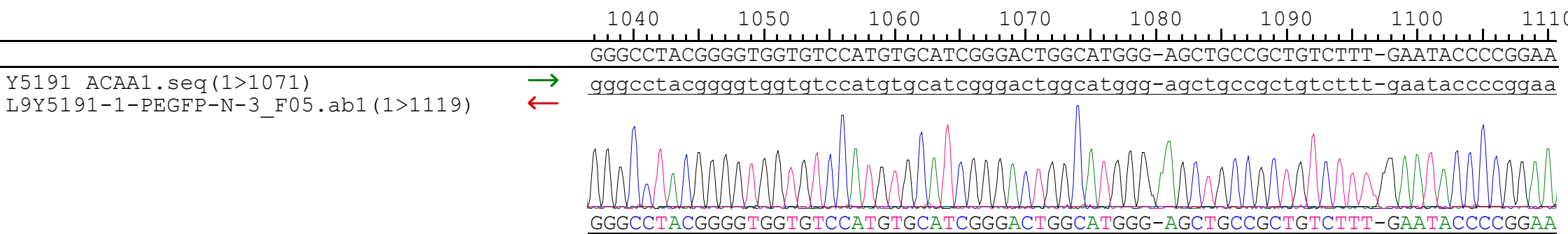

1120 1130 1140 1150 1160

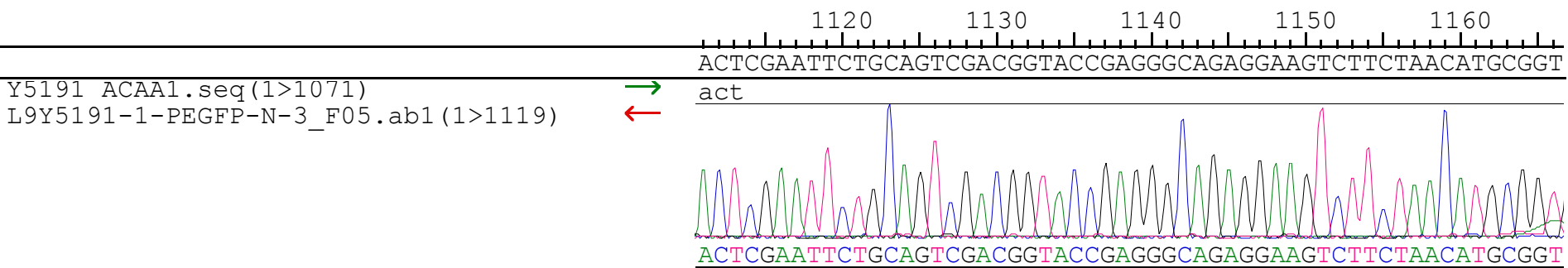

Supplement: Supplementary file 3 [file Data_Sheet_3.PDF]
